# Comparison between Intravitreal Triamcinolone with Grid Laser Photocoagulation versus Bevacizumab with Grid Laser Photocoagulation Combinations for Branch Retinal Vein Occlusion

**DOI:** 10.1155/2013/141279

**Published:** 2013-12-19

**Authors:** Abdullah Ozkaya, Ugur Celik, Zeynep Alkin, Miray Faiz Turan, Ahmet Taylan Yazici, Ahmet Demirok

**Affiliations:** ^1^Beyoglu Eye Training and Research Hospital, Bereketzade Camii Sok., Kuledibi, Beyoglu, 34421 Istanbul, Turkey; ^2^Department of Ophthalmology, Medeniyet University, 34700 Istanbul, Turkey

## Abstract

*Purpose*. To compare the efficacy of intravitreal triamcinolone (IVT) and intravitreal bevacizumab (IVB), both combined with grid laser photocoagulation (GLP) for macular edema (ME) secondary to branch retinal vein occlusion (BRVO). *Methods*. Retrospective, comparative study. The newly diagnosed patients with ME secondary to BRVO who were treated with IVT and GLP or IVB and GLP were included. The main outcome measures were changed in the best corrected visual acuity (BCVA) and central retinal thickness (CRT) from the baseline to month 24. *Results*. Ninety-nine eyes of 99 patients were included. The change in BCVA was not statistically different in any time points between the two groups (*P* > 0.05, for all). The change in CRT was not statistically different in any time points between the two groups (*P* > 0.05, for all). The mean number of injections at month 24 was 2.38 ± 1.06 in the IVT+GLP group and 4.17 ± 1.30 in the IVB+GLP group (*P* = 0.0001). The need for cataract surgery (*P* = 0.01) and secondary glaucoma (*P* = 0.03) occurrence were more common in IVT group. * Conclusion*. Both treatment modalities were effective in the treatment of ME secondary to BRVO. The number of injections was significantly lower in the IVT group than in the IVB group; however cataract and secondary glaucoma were more frequent in the IVT+GLP group than in the IVB+GLP group.

## 1. Introduction

Branch retinal vein occlusion (BRVO) is the second most common cause of retinal vascular disease following diabetic retinopathy [1–3]. Among the changes that define BRVO, macular edema (ME) is a frequent cause of visual acuity loss [2, 3]. Grid laser photocoagulation (GLP) is the only proven long-term effective therapy for ME secondary to BRVO [3]. In the Branch Vein Occlusion Study, it is shown that GLP results in a significant improvement in vision in 65% of the patients; however, the clinical outcomes are sometimes disappointing [3]. Therefore, during the last decade, several studies support the use of intravitreal pharmacotherapies as adjuncts or alternative treatments to laser photocoagulation [4]. Intravitreal corticosteroid and antivascular growth factor (VEGF) injections have been widely investigated in ME secondary to BRVO. Several reports indicated that intravitreal triamcinolone (IVT) injection is an efficacious therapy to prevent the patients with ME secondary to BRVO from loss of vision and retinal thickening [5–9]. Intravitreal bevacizumab (IVB) injection is another treatment option [10, 11]. As an anti-VEGF agent, intravitreal bevacizumab blocks the effects of VEGF, which include increased vascular permeability and subsequent ME [10, 11]. The beneficial effects of intravitreal anti-VEGF drugs have been suggested for the reduction of ME from different etiologies, including BRVO [12–14]. In addition, intravitreal injection of ranibizumab, a monoclonal antibody fragment that inhibits VEGF, and an intravitreal dexamethasone implant (Ozurdex) are the other therapeutic options for BRVO [15].

Intravitreal triamcinolone injection on a basis of an as-needed regimen every four months and monthly anti-VEGF injections were both found to be effective in treating ME secondary to BRVO [15]. However, both of the treatment regimens have some limitations. The effectiveness of IVT injection was not maintained after 1 year, despite repeated injections with high cataract and glaucoma rates [15]. The need for repeated injections, the potential cardiovascular side effects of anti-VEGF agents, and the upregulation of the VEGF receptors due to repeated injections are the limitations of anti-VEGF therapy [15]. Therefore, combined therapies may be a good option for the treatment of ME secondary to BRVO. The combination of IVT injection with GLP and the combination of intravitreal bevacizumab (IVB) injection with GLP have been proposed and have obtained positive outcomes [16, 17]. In the literature the efficacy of IVB monotherapy versus IVT combination with GLP in the treatment of diffuse diabetic macular edema was studied [18]. However, a literature search (from PubMed and MEDLINE search) revealed that there was no study that compared the combinations of IVT with GLP and IVB with GLP for the treatment of ME secondary to BRVO. The purpose of this retrospective study was to compare the visual and anatomical outcomes of IVT combined with GLP and IVB combined with GLP in ME secondary to BRVO.

## 2. Methods

This retrospective, comparative, and interventional study included the patients with macular edema secondary to BRVO, who underwent IVT injections combined with GLP or IVB injections combined with GLP between January 2008 and January 2012. The newly diagnosed BRVO patients who had macular edema since <3 months at the first admission and who were treatment naive for BRVO were included in the study. The patients who had coexisting retinal disease (like diabetic retinopathy and epiretinal membrane), media opacities that could decrease visual acuity, or macular ischemia were not included. The tenets of the Declaration of Helsinki were followed throughout the study, and written informed consent was obtained from all patients for the treatments.

### 2.1. Data

Data collected from the patients' records included age, gender, type of BRVO, BCVA, and central retinal thickness (CRT) before treatment at months 3, 6, 9, 12, 15, 18, 21, and 24. Cumulative numbers of injections were recorded for each patient.

### 2.2. Examinations

All patients underwent a standardized examination including measurement of BCVA via the early treatment diabetic retinopathy study (ETDRS) chart at 4 meters, slit-lamp biomicroscopy, and fundus examination and measurement of intraocular pressure (IOP) via applanation tonometry. Fundus photography, fluorescein angiography (HRA-2, Heidelberg Engineering, Heidelberg, Germany), and optical coherence tomography (OCT) imaging (Stratus OCT TM, Carl Zeiss Meditec Inc., Dublin, CA, USA) were performed before treatment. All examinations were repeated at all of the visits, except fluorescein angiography, which was repeated only when the cause of visual acuity deterioration could not be clarified with the clinical examination and other imaging methods. OCT was used for the measurement of CRT, that being defined as the mean thickness of the neurosensory retina in the central 1 mm diameter region, computed via OCT mapping software provided with the device. Fluorescein angiography was inspected for capillary dropout zones at the fovea and peripheral retina and leaking areas, which were decided to be the cause of ME. The type of the disease was defined as ischemic BRVO, if there was an ischemic area, which was ≥5 disc areas at the posterior pole; the patients were not included in the study.

### 2.3. Injection Method

All injections were performed under sterile conditions after topical anesthesia and 10% povidone-iodine scrub (Betadine, Purdue Pharma, Stamford, CT, USA) was used on the lids and lashes, and then 5% povidone-iodine was administered on the conjunctival sac. Intravitreal injection of 4 mg/0.1 mL of triamcinolone (Kenacort-A 40 mg/mL, Bristol-Myers Squibb Co., Princeton, NJ) and 1.25 mg/0.05 mL of bevacizumab (Avastin, Genentech Inc., South San Francisco, CA, USA) was injected with a 27-gauge needle through the pars plana at 3.5 mm to 4 mm posterior to the limbus. After the intravitreal injections, an ophthalmic solution of topical levofloxacin was administered 5 times a day for a week and the patients were then instructed to consult the hospital if they experienced decreased vision, eye pain, or any new symptoms.

### 2.4. Grid Laser Photocoagulation Method

Grid laser photocoagulation was performed over the focal leaks seen on the FA and on areas of diffuse retinal thickening with a 532 diode-pumped solid-state laser (Visulas 532s, Zeiss-Humphrey systems, Carl Zeiss, Jena, Germany). The settings used for GLP were as follows: spot diameter, 100 *μ*m; exposure time, 0.1 seconds; and power 50–150 mW. The settings were adjusted to be powerful enough to create a soft whitening of the retina, according to the discretion of the physician. Grid laser photocoagulation was applied 1 month after the first IVT or IVB injection in all cases. After the first GLP treatment the patients were assessed for the need for additional GLP retreatment for 4-month intervals and additional GLP was applied when new focal leaks or areas of diffuse retinal thickening were detected via FA.

### 2.5. Treatment Schedule

Initially, all patients received an IVT or IVB injection, followed by GLP after 1 month. Both groups were followed up monthly and further reinjections were planned according to as-needed treatment strategy based on the following criteria: ≥1 lines of visual acuity loss or any increase in CRT in OCT images. Patients were retreated at 4-month intervals in the IVT group and monthly in the IVB group when at least one of the retreatment criteria was met.

### 2.6. Main and Secondary Outcome Measures

The main outcome measures of this study were changed in BCVA and CRT from the baseline to month 24 followup visit. The Secondary outcome measures were the number of injections, and complication rates between the IVT and IVB groups.

### 2.7. Data Analysis

Visual acuity was converted to logarithm of minimum angle of resolution (LogMAR) for statistical analysis. The change in BCVA and CRT over time was analyzed with paired sample *t*-test. Chi-square test was used to compare nominal parameters between the groups and independent sample *t*-test was used for continuous parameters. The statistical analysis was performed using SPSS (version 15.0, SPSS Inc., Chicago, IL, USA). A *P* value of less than 0.05 was considered to be statistically significant.

## 3. Results

Ninety-nine eyes of 99 patients were included in the study. Fifty-two patients were treated with IVT plus GLP and 47 patients were treated with IVB plus GLP. The baseline general characteristics were similar between the two groups (Table 1).

Mean baseline, month 12, and month 24 BCVA was 0.72 ± 0.51 LogMAR (range 1.8–0.3 LogMAR), 0.59 ± 0.43 LogMAR (range 1.8–0.0 LogMAR), and 0.57 ± 0.43 LogMAR (range 1.8–0.0 LogMAR), respectively, in the IVT group. Mean baseline, month 12, and month 24 BCVA was 0.71 ± 0.50 LogMAR (range 1.8–0.3 LogMAR), 0.60 ± 0.47 LogMAR (range 1.5–0.0 LogMAR), and 0.57 ± 0.63 LogMAR (range 1.3–0.0 LogMAR), respectively, in the IVB group. The change in mean BCVA from baseline to months 3, 6, 9, 12, 15, 18, 21, and 24 was statistically better in both of the groups (*P* < 0.05 for all). However, there was not a statistically significant difference between the two groups in regard of change in BCVA at all of the time points (*P* > 0.05 for all) (Table 2). At month 24, 18 of the 52 patients (34.6%) in the IVT group and 16 of the 47 patients (34.0%) in the ranibizumab group gained VA ≥ 3 lines (*P* = 0.9).

Mean baseline, month 12, and month 24 CRT was 491 ± 119 microns (range 239–764 microns), 251 ± 68 microns (range 174–648 microns), and 227 ± 61 microns (range 154–537 microns), respectively, in the IVT group. Mean baseline, month 12, and month 24 CRT was 520 ± 165 microns (range 188–810 microns), 283 ± 71 microns (range 187–456 microns), and 258 ± 78 microns (range 150–514 microns), respectively. The change in mean CRT from baseline to month 3, 6, 9, 12, 15, 18, 21, and 24 was statistically better in both of the groups (*P* < 0.0001 for all). However, there was not a statistically significant difference between the two groups in regards of change in CRT at all of the time points (*P* > 0.05 for all) (Table 3).

The mean number of injections at month 12 was 1.6 ± 1.3 (range 1–4) in the IVT group and 2.5 ± 0.8 (range 1–4) in the IVB group (*P* = 0.001), and the total number of injections at month 24 was 2.3 ± 1.0 (range 1–6) in the IVT group and 4.1 ± 1.3 (range 2–7) in the IVB group (*P* = 0.0001). The mean number of GLP treatments at month 24 was 1.4 in the IVT group and 1.5 in the IVB group (*P* = 0.6).

Complications were listed in Table 4. Secondary glaucoma and cataract formation were observed more frequently in the IVT group.

## 4. Discussion

The prevalence of retinal venous occlusion has been found to be between 0.7% and 1.6% [19–22]. Retinal vein occlusion has two main types: central retinal vein occlusion (CRVO) and frequently seen BRVO, which was first reported in 1877 [21]. BRVO has two main types: major BRVO, which is related to the occlusion of one of the major branch retinal veins, and macular BRVO, which is related to the occlusion of one of the macular venules [3]. In major branch BRVO, 66% of superotemporal quadrant and 22–43% of inferotemporal quadrant were found to be occluded [22]. In this study we found the superotemporal/inferotemporal ratios to be similar to the literature in both groups.

Branch retinal vein occlusion presents with painless vision loss [22] and usually occurs at the sites where arterioles cross over veins [3, 15]. Fluorescein angiography is important for diagnosis, providing the type of RVO, and defining the condition of macula concerning the ME and macular ischemia [23]. Optical coherence tomography provides additional information for the assessment of retinal thickness and the location of ME [24].

Branch retinal vein occlusion treatment is based on the management of ME, retinal neovascularization, vitreous hemorrhage, and tractional retinal detachment, which are the most common complications of BRVO [22].

Grid laser photocoagulation has been shown to improve the oxygenation of the inner retina [3, 23, 25, 26]. The oxygen consumption of photoreceptors decreases at the outer retina; this allows oxygen diffusion from the choroid to the inner retina, which increases the oxygen tension and relieves hypoxia after GLP [26–28]. Inner retina oxygenation allows autoregulatory vasoconstriction and leads to reduced hydrostatic pressure in the capillaries and venules [25]. Eventually this decreases the fluid effusion and reduces the edema in the retinal tissues. In the prospective randomized study by the Branch Vein Occlusion Study Group it was reported that GLP significantly improved long-term visual prognosis in ME caused by BRVO [7]. In the same study it was reported that the patients with a visual acuity of 20/40 or less showed a significant benefit compared with the control group [7].

The aqueous humor level of proinflammatory cytokines such as interleukin and VEGF was found to be elevated in RVO patients, which was significantly correlated with the severity of macular edema. These findings suggest that inflammation may contribute to the development and progression of ME in patients with RVO [5, 10, 15, 26, 27]. Intravitreal anti-VEGF injection is an alternative therapy for the BRVO patients. Campochiaro et al. [28] first reported the efficacy of IVB for ME secondary to BRVO. Many studies showed the benefit of IVB treatment with an improvement in visual acuity and a decrease of retinal thickness in BRVO patients [29–31]. Another therapy for the BRVO patients is intravitreal corticosteroids, which inhibit not only VEGF but also various proinflammatory mediators that contribute to the pathogenesis of RVO [1, 32]. However, IVT treatment could only show the stabilization or a moderate improvement in visual acuity [33–35]. Furthermore, IVT injection has side effects, such as cataract formation or increased intraocular pressure, which are not commonly seen in IVB injections [30, 31, 35]. The standard care versus corticosteroid for retinal vein occlusion (SCORE) study group reported that, after 12 months and through 36 months, GLP monotherapy was superior to both 1 mg and 4 mg IVT monotherapies by means of visual acuity improvement and decrease in CRT [36]. In the SCORE study, the mean improvement in visual acuity score from baseline to month 36 was 12.9 letters in GLP group, 4.4 letters in 1 mg IVT group, and 8.0 letters in 4 mg IVT group [36]. The median decrease in CRT from baseline to month 36 was 312, 245, and 250 *μ* in GLP, 1 mg IVT, and 4 mg IVT groups, respectively [36]. In addition, the GLP group was superior to both IVT groups, according to the need for antiglaucomatous drugs and progression of lens opacity [36]. A dexamethasone intravitreal implant (DEX implant) (Ozurdex) is approved by US Food and Drug Administration for the therapy of ME secondary to RVOs [15]. Phase III study of DEX implant was designed to evaluate the efficacy and safety of 1 or 2 treatments of DEX implant over 12 months in eyes with macular edema secondary to branch BRVO or central retinal vein occlusion. The study showed that mean BCVA improvement was significantly greater in dexamethasone intravitreal implant than sham group at days 30 to 90 [15]. However, this improvement became nonsignificant after 3 months. After the second injection, which was performed at month 6, the results were again favorable only from month 6 to month 9, and the beneficial effect of DEX implant did not last up to month 12. The implant was designed to cause fewer side effects than triamcinolone; however, 25% of the patients showed intraocular pressure rise which peaked at day 60 and returned to baseline by day 180 and cataract progression was seen in 26% of patients after 1 year [15].

Bevacizumab was the first anti-VEGF drug reported to show efficacy in the resolution of ME secondary to RVOs [28]. In a prospective study by Prager et al. [37], monthly IVB injections according to an as-needed treatment regime were found to be effective in ME secondary to BRVO. In the study it is reported that, after 12 months of follow-up time, BCVA was found to be increased by 18 letters, the mean decrease in CRT was 241 *μ*m, and the patients received a mean of 8 injections. In addition, no severe ocular or systemic adverse events were reported in the study [38]. Ranibizumab is another anti-VEGF agent, which is a monoclonal antibody fragment derived from the same parent antibody as bevacizumab [37]. In a large multicenter prospective study, Ranibizumab for the Treatment of Macular Edema following Branch Retinal Vein Occlusion (BRAVO), monthly 0.3 mg and 0.5 mg intravitreal ranibizumab injections were found to be superior to sham injections in the first 6 months [39]. After 6 months, the patients were treated on an as-needed injection regimen. At month 12, the mean improvement from baseline in ETDRS letter score was 16.4 letters in 0.3 mg ranibizumab group and 18.1 letters in 0.5 mg ranibizumab group. The mean number of injections was 8.9 in 0.3 mg ranibizumab group and 8.8 in 0.5 ranibizumab group after 12 months of followup time [40]. Salinas-Alamán et al. [17] evaluated the effect of bevacizumab associated with grid laser in ME secondary to BRVO. In the study, IVB was administered at baseline followed by GLP 1 month after. Then during the followup period additional IVB injections were administered on an as-needed regimen. After a followup period of 12 months, mean BCVA was found to be increases from 0.28 to 0.66 (Snellen lines), mean CRT was found to be decreased from 479 microns to 335 microns, and the mean injection number was found to be 2.13. In a similar study by Donati et al. [2], IVB monotherapy was compared with IVB combined with GLP in patients with ME secondary to BRVO. After a followup period of 12 months, the mean BCVA increased from 0.7 to 0.4 LogMAR in the IVB monotherapy group and increased from 0.6 to 0.2 LogMAR in the combined therapy group. The mean CRT decreased from 420 to 323 microns in the IVB monotherapy group and decreased from 386 to 238 microns in the combined therapy group. The mean number of injections was reported as 4 in the monotherapy group and 3 in the combined therapy group. In our study, the mean injection numbers were found to be very low when compared with those in the other studies: 2.3 injections in IVT group and 4.1 injections in IVB group over 24 months. The adjuvant, long-lasting effect of GLP alleviated the need for repeated injections. Even the GLP reduces angiographic ME in eyes with BRVO; it may be difficult to perform effective GLP when ME is severe. In these cases, it is often necessary to increase the laser power in order to ensure sufficient coagulation. By combining IVB and IVT with GLP, we produced burns with a relatively low setting of the laser (50–150 mW in intensity, 0.1 millisecond in duration, and 100 *μ*m in spot size). This protocol allowed us to perform GLP more safely. In our study all of the patients underwent only one session of GLP during followup, and GLP was applied 1 month after the first IVB or IVT injection in all cases. In addition, the retreatment criteria of our study were different from those of the other study. There was not a cutoff level for CRT thickness in the retreatment criteria. We retreated the patients only when we have detected ≥1 lines of visual acuity loss or any increase in CRT in OCT images. The treatment target was mainly based on visual acuity loss and decreasing the CRT but not absolutely drying the macula; therefore, the mean injection numbers were low in our study.

In this study, a significant improvement in BCVA and a decrease in mean CRT were achieved in both groups. There was not a significant difference between the two groups in regard of visual and anatomical outcomes. In the comparison studies of bevacizumab and triamcinolone monotherapies for the treatment of ME secondary to BRVO, both were found to be effective for ME with decreased CRT values [29, 34]. Rosenfeld et al. [29] reported that IVB injection was more effective than IVT injection for the treatment of ME secondary to BRVO without ocular and systemic complications.

In this study, epiretinal membrane formation was detected in both groups; glaucoma and cataract formation were detected only in the IVT group. During the followup period, no severe ocular adverse effects (such as endophthalmitis, retinal detachment, traumatic cataract, uveitis, and central retinal artery occlusion) were observed in any of the groups.

The main limitation of the study is its retrospective design, relatively low patient number, and the retreatment criteria of the study which did not include a criterion for changes in CRT. A prospective study including large group of patients may be designed to include four groups: IVT monotherapy, IVT combined with GLP, IVB monotherapy, and IVB combined with GLP. These groups may evaluate the contribution of GLP therapy to intravitreal injections and better delineate the efficacy of the two drugs in the treatment of ME secondary to BRVO.

In summary, both IVT and IVB injections combined with GLP therapy appeared to be effective treatment options for ME secondary to BRVO both with low number of injections. The IVT injections may cause several side effects like cataract formation and glaucoma; therefore, IVB injections may be preferred instead of IVT in this group of patients.

## Figures and Tables

**Table 1:** General characteristics of the patients.

	IVT + GLP	IVB + GLP	*P* value
Number of eyes	52	47	—
Age (years)	62.8 ± 8.4	64.6 ± 8.7	*P* = 0.48
Gender (male/female)	32/20	28/19	*P* = 0.42
Hypertension (%)	35 (67.3%)	26 (55.4%)	*P* = 0.18
Diabetes (%)	12 (22.8%)	11 (23.6%)	*P* = 0.27
Hyperlipidemia (%)	4 (7.6%)	4 (8.6%)	*P* = 0.41
Fluorescein angiography (nonischemic/ischemic)	43/9	30/17	*P* = 0.29
Localization of BRVO (superotemporal/inferotemporal)	38/14	31/16	*P* = 0.48
Initial IOP (mmHg)	15.0 ± 2.0	15.2 ± 2.0	*P* = 0.89
Last IOP (mmHg)	16.0 ± 1.8	15.1 ± 2.4	*P* = 0.12

IVA: intravitreal triamcinolone; GLP: grid laser photocoagulation; IVB: intravitreal bevacizumab; *P*: *P* value; BRVO: branch retinal vein occlusion; IOP: intraocular pressure. *P value less than 0.05 was considered statistically significant. *P* values are for IVT versus IVB groups.

**Table 2:** The mean visual acuity levels and the change in visual in the study groups at different time points.

	IVT + GLP	IVB + GLP	*P* values*
Baseline BCVA (LogMAR)	0.72 ± 0.51	0.71 ± 0.50	*P* = 0.9

Month 3 BCVA (LogMAR)	0.58 ± 0.38	0.60 ± 0.43	*P* = 0.6
Mean change from baseline (LogMAR)	0.13 ± 0.35	0.11 ± 0.26	
Baseline versus month 3 *P* value	*P* = *0.006 *	*P* = *0.007 *	

Month 6 BCVA (LogMAR)	0.56 ± 0.40	0.59 ± 0.45	*P* = 0.6
Mean change from baseline (LogMAR)	0.16 ± 0.47	0.12 ± 0.33	
Baseline versus month 6 *P* value	*P* = *0.01 *	*P* = *0.01 *	

Month 9 BCVA	0.57 ± 0.42	0.60 ± 0.46	*P* = 0.6
Mean change from baseline (LogMAR)	0.15 ± 0.46	0.11 ± 0.34	
Baseline versus month 9 *P* value	*P* = *0.02 *	*P* = *0.03 *	

Month 12 BCVA (LogMAR)	0.59 ± 0.43	0.60 ± 0.47	*P* = 0.8
Mean change from baseline (LogMAR)	0.13 ± 0.49	0.11 ± 0.38	
Baseline versus month 12 *P* value	*P* = *0.03 *	*P* = *0.04 *	

Month 15 BCVA (LogMAR)	0.59 ± 0.44	0.59 ± 0.45	*P* = 0.9
Mean change from baseline (LogMAR)	0.13 ± 0.50	0.12 ± 0.38	
Baseline versus month 15 *P* value	*P* = *0.04 *	*P* = *0.03 *	

Month 18 BCVA (LogMAR)	0.58 ± 0.43	0.59 ± 0.45	*P* = 0.8
Mean change from baseline (LogMAR)	0.14 ± 0.51	0.12 ± 0.39	
Baseline versus month 18 *P* value	*P* = *0.03 *	*P* = *0.03 *	

Month 21 BCVA (LogMAR)	0.57 ± 0.43	0.57 ± 0.46	*P* = 0.9
Mean change from baseline (LogMAR)	0.15 ± 0.52	0.14 ± 0.39	
Baseline versus month 21 *P* value	*P* = *0.03 *	*P* = *0.01 *	

Month 24 BCVA (LogMAR)	0.57 ± 0.43	0.57 ± 0.63	*P* = 0.9
Mean change from baseline (LogMAR)	0.15 ± 0.51	0.14 ± 0.39	
Baseline versus month 24 *P* value	*P* = *0.03 *	*P* = *0.02 *	

*for baseline visual acuity, *P* values for IVT versus IVB are for the baseline values themselves; the other *P* values are for the change achieved with the two drugs relative to the baseline values. BCVA: best corrected visual acuity; LogMAR: logarithm of the minimal angle of resolution; IVT: intravitreal triamcinolone; GLP: grid laser photocoagulation; IVB: intravitreal bevacizumab; *P value less than 0.05 was considered statistically significant.

**Table 3:** The mean central retinal thickness values and the change in central retinal thickness in the study groups at different time points.

	IVT + GLP	IVB + GLP	*P* value*
Baseline CRT (microns)	491 ± 119	520 ± 165	0.3

Month 3 CRT (Microns)	313 ± 101	363 ± 165	0.3
Mean change from baseline (Microns)	178 ± 95	157 ± 114	
Baseline vs month 3 *P* value	*P* < *0.0001 *	*P* < *0.0001 *	

Month 6 CRT (Microns)	281 ± 79	294 ± 81	0.4
Mean change from baseline (Microns)	209 ± 89	226 ± 128	
Baseline vs month 6 *P* value	*P* < *0.0001 *	*P* < *0.0001 *	

Month 9 CRT	261 ± 66	288 ± 68	0.9
Mean change from baseline (Microns)	229 ± 121	232 ± 162	
Baseline vs month 9 *P* value	*P* < *0.0001 *	*P* < *0.0001 *	

Month 12 CRT (Microns)	251 ± 68	283 ± 71	0.9
Mean change from baseline (Microns)	239 ± 117	237 ± 148	
Baseline vs month 12 *P* value	*P* < *0.0001 *	*P* < *0.0001 *	

Month 15 CRT (Microns)	255 ± 54	275 ± 78	0.7
Mean change from baseline (Microns)	235 ± 115	245 ± 155	
Baseline vs month 15 *P* value	*P* < *0.0001 *	*P* < *0.0001 *	

Month 18 CRT (Microns)	236 ± 39	271 ± 69	0.8
Mean change from baseline (Microns)	254 ± 112	249 ± 155	
Baseline vs month 18 *P* value	*P* < *0.0001 *	*P* < *0.0001 *	

Month 21 CRT (Microns)	235 ± 56	265 ± 77	0.9
Mean change from baseline (Microns)	256 ± 113	255 ± 146	
Baseline vs month 21 *P* value	*P* < *0.0001 *	*P* < *0.0001 *	

Month 24 CRT (Microns)	227 ± 61	258 ± 78	0.9
Mean change from baseline (Microns)	263 ± 129	262 ± 153	
Baseline vs month 24 *P* value	*P* < *0.0001 *	*P* < *0.0001 *	

*for baseline CRT, *P* values for IVT versus IVB are for the baseline values themselves; the other *P* values are for the change achieved with the two drugs relative to the baseline values. CRT: central retinal thickness; vs: versus; IVT: intravitreal triamcinolone; GLP: grid laser photocoagulation; IVB: intravitreal bevacizumab; *P value less than 0.05 was considered statistically significant.

**Table 4:** Complications in the study groups.

	IVT + GLP	IVB + GLP	*P *
Uveitis	3 patients	3 patients	0.9
Subconjunctival hemorrhage	4 patients	4 patients	0.9
Epiretinal membrane	8 patients	7 patients	0.8
Glaucoma (required chronic medication)	3 patients	0 patients	*0.04 *
Cataract surgery requirement during the study period	11 patients	4 patients	*0.02 *

IVT: intravitreal triamcinolone; GLP: grid laser photocoagulation; IVB: intravitreal bevacizumab; *P*: *P* value. *P value less than 0.05 was considered statistically significant. *P* values are for IVT versus IVB groups.
